# Supplementary material for: Polymeric Nanoparticles Enable mRNA Transfection and Its Translation in Intervertebral Disc and Human Joint Cells, Except for M1 Macrophages
Source: Pharmaceutics. 2024 Mar 22;16(4):438. doi: 10.3390/pharmaceutics16040438 (PMC11053495; doi:10.3390/pharmaceutics16040438)
Supplement: Supplementary file 1 [file pharmaceutics-16-00438-s001.zip › pharmaceutics-2903320-supplementary.pdf]

Polymeric nanoparticles enable mRNA transfection and its translation in intervertebral disc and human joint cells, except for M1 macrophages

*Laura B. Creemers is the corresponding author; she can be reached by following email address: l.b.creemers@umcutrecht.nl

Supplementary figures

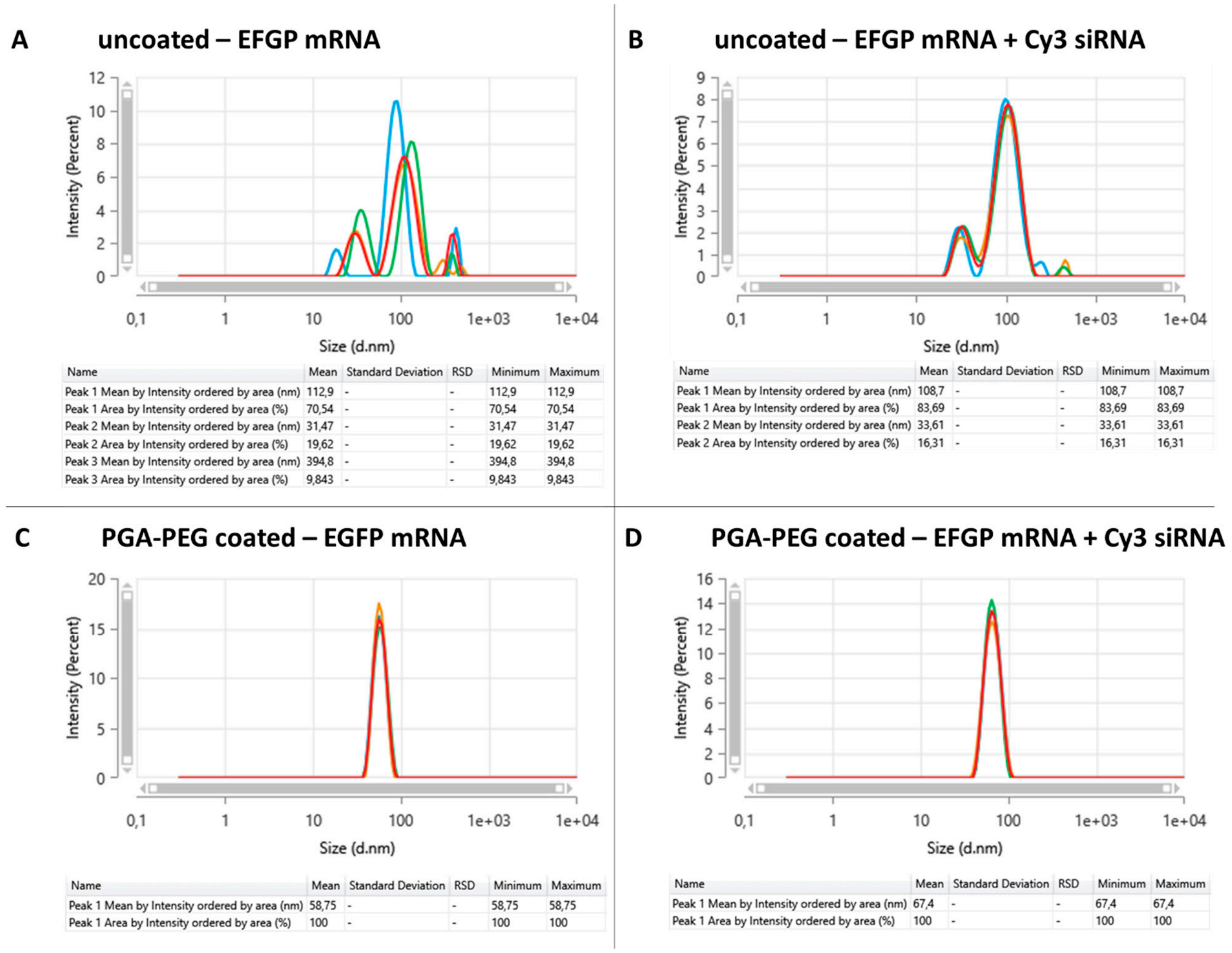

A

Name	Mean	Standard Deviation	RSD	Minimum	Maximum
Peak 1 Mean by Intensity ordered by area (nm)	112,9	-	-	112,9	112,9
Peak 1 Area by Intensity ordered by area (%)	70,54	-	-	70,54	70,54
Peak 2 Mean by Intensity ordered by area (nm)	31,47	-	-	31,47	31,47
Peak 2 Area by Intensity ordered by area (%)	19,62	-	-	19,62	19,62
Peak 3 Mean by Intensity ordered by area (nm)	394,8	-	-	394,8	394,8
Peak 3 Area by Intensity ordered by area (%)	9,843	-	-	9,843	9,843

B

Name	Mean	Standard Deviation	RSD	Minimum	Maximum
Peak 1 Mean by Intensity ordered by area (nm)	108,7	-	-	108,7	108,7
Peak 1 Area by Intensity ordered by area (%)	83,69	-	-	83,69	83,69
Peak 2 Mean by Intensity ordered by area (nm)	33,61	-	-	33,61	33,61
Peak 2 Area by Intensity ordered by area (%)	16,31	-	-	16,31	16,31

C

Name	Mean	Standard Deviation	RSD	Minimum	Maximum
Peak 1 Mean by Intensity ordered by area (nm)	58,75	-	-	58,75	58,75
Peak 1 Area by Intensity ordered by area (%)	100	-	-	100	100

D

Name	Mean	Standard Deviation	RSD	Minimum	Maximum
Peak 1 Mean by Intensity ordered by area (nm)	67,4	-	-	67,4	67,4
Peak 1 Area by Intensity ordered by area (%)	100	-	-	100	100

Figure S1: Multi-angle Dynamic Light Scattering (MADLS) data for all nanoparticle types investigated. Red curve is the combined data of three angles, 175° (blue), 90° (orange) and 13° (green), with three individual measurements per angle. For A, the table shows that for Peak 1, 70.54% of the readouts fall within the mean particle size of 112.9 nm. For Peak 2, 19.62% of the measurements fall within the mean diameter of 31.47 nm, and so on. Uncoated nanoparticles A and B are not monodisperse, as multiple peaks are seen. For PGA-PEG coated nanoparticles C and D, we can assume monodispersity as 100% of the measurements fall within a single peak of intensity values from the tables refer only to the <u>*red curve*</u> *in the size distribution.*

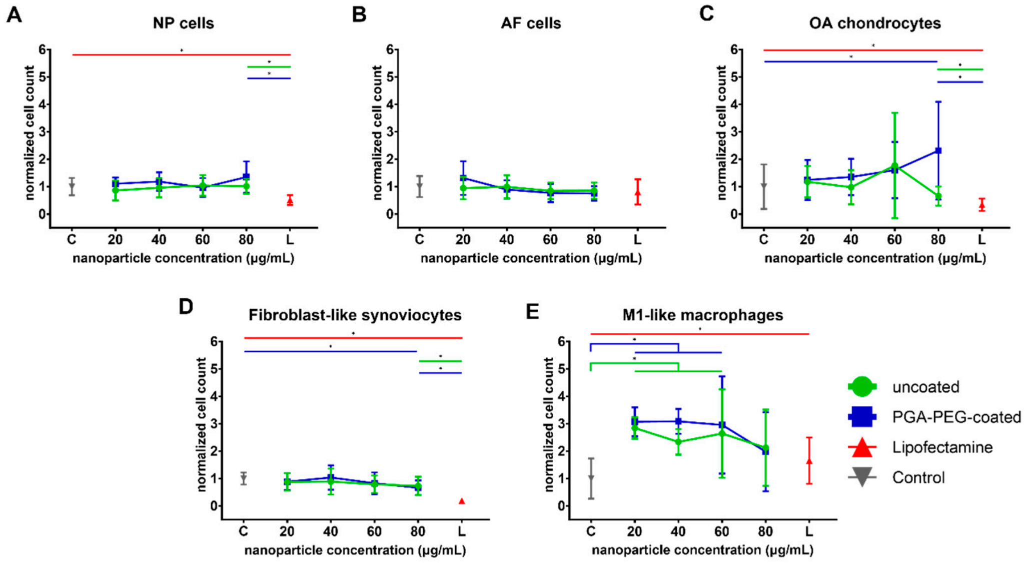

Figure S2: Cell count determined as Hoechst-positive cells for A: NP cells, B: AF cells, C: OA chondrocytes, D: fibroblast-like synoviocytes and E: M1-like macrophages. mRNA concentration of Lipofectamine control (red) is 3.2 µg/mL. Values were normalized to control.

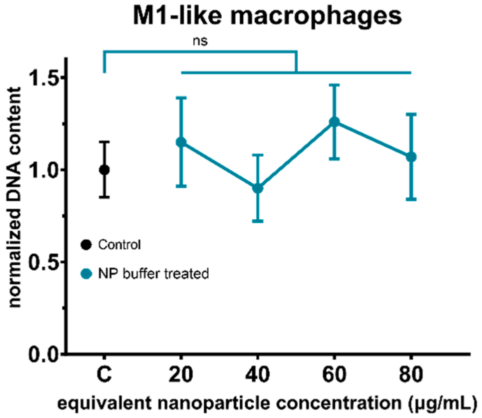

Figure S3: PicoGreen analysis of M1-like macrophages treated with nanoparticle buffer. Concentrations of buffer are equivalent to the buffer concentrations used in the dose response experiments using nanoparticles in buffer. Data of 6 replicates from 1 donor.
